# East Meets West: A Multisite Validity Study of the China Medical Professionalism Inventory

**DOI:** 10.5334/pme.1682

**Published:** 2025-09-25

**Authors:** Honghe Li, David A. Hirsh, Xinzhi Song, Edward Krupat, Xue Yang, Ming-Jung Ho, Dianne Manning, Deliang Wen

**Affiliations:** 1 Institute of Health Professions Education Assessment and Reform, School of Medical Humanities, China Medical University, Shenyang, Liaoning, China; 2 Department of Medicine at Harvard Medical School, Boston Massachusetts and Cambridge Health Alliance, Cambridge, Massachusetts, USA; 3 Institute of Health Professions Education Assessment and Reform, China Medical University, Shenyang, Liaoning, China; 4 Center for Evaluation (retired), Harvard Medical School, Massachusetts, USA; 5 Foundation for Advancement of International Medical Education and Research, Philadelphia, PA, USA; 6 Faculty of Health Sciences, University of Pretoria, Pretoria, South Africa

## Abstract

**Introduction:**

The characteristics of medical professionalism (MP) vary across cultural contexts. Professionalism constructs and MP tools currently rely on Western cultural perspectives. Chinese leaders are calling for MP tools that connect to historical traditions, current culture, and modern conceptualizations of MP inside and outside China.

**Methods:**

The authors developed the China Medical Professionalism Inventory using standard processes in two steps. Phase I, “development of item pool,” involved reviewing the literature to generate an item pool and conducting a first survey of Chinese clinical experts to develop content evidence. Phase II, “delineation of validity evidence,” included three psychometric studies of practicing physicians and a second expert survey to create the final version of the tool; these processes aimed to determine validity evidence for content, internal structure, and relationships to other variables.

**Results:**

Systematic review of the English- and Chinese-language literature identified 1537 professionalism-specific items from 63 sources to form the item pool. The authors conducted two rounds of expert review, including surveying nationally prominent Chinese clinician-leaders (n = 34, response rate 85%, and n = 76, response rate 63%). The authors conducted three psychometric studies of practicing Chinese physicians (n = 360, response rate 92%; n = 3653, response rate 90%; and n = 955, response rate 95%). The results generated the 20-item CMPI, with four factors: “Respect, Compassion, and Communication; Integrity; Excellence; and Responsibility.”

**Discussion:**

The CMPI presented validity evidence for content, internal structure, and relationship to other variables. This study may extend the conceptualization and reach of MP measurement.

## Introduction

Medical professionalism (MP) is a complex, multi-dimensional construct [1,2] that fosters professional relationships, promotes public trust, and enhances patient safety [3,4]. Elements and conceptualizations of MP connect to social and cultural contexts [5,6,7,8,9,10,11]. To train and assess physicians and health care professionals, educators and leaders require culturally informed and contextually aligned MP tools.

Researchers report cultural variations in behaviors viewed as professional [5,6,7,8,9,10,11], and in China, educators and researchers are now advocating for an MP tool that can connect to historical tradition, current culture, and modern conceptualizations of MP inside and outside China [6,12]. Multiple reviews highlight that current MP tools embody Western frameworks and were developed in Western contexts [7,13,14,15]. These tools are translated for MP assessment in Eastern contexts [12,13], a one-way process that may not account for the diversity, contextual specificity, and cultural expectations of MP.

In our increasingly multipolar world, cultures, ideologies, and values intertwine and shape diverse aspects of society, including medicine [11]. Western conceptualizations have profoundly impacted modern MP, but this does not imply that Western conceptualizations fully account for voices, values, and practices in other cultures [11]. Aligning with traditional Chinese philosophical principles of “inclusiveness” (兼容并蓄, jiān róng bìng xù) and "harmony in diversity" (和而不同, hé ér bù tóng), this study aimed to develop an MP tool that incorporated Chinese values and cultural foundations while embracing international perspectives. This goal remains elusive, even as researchers have made progress defining MP in non-Western contexts [11,16,17,18,19,20,21].

The literature reports more than 80 instruments related to MP [13,22,23,24,25,26,27,28,29,30]. Systematic reviews evaluating psychometric properties of MP tools suggest that only a limited number of studies document acceptable evidence of reliability and validity [12,13,14,15]. Ideally, MP tool development should follow standard processes for psychological testing [31,32,33,34,35] and report evidence for validity incorporating the context of the tool’s use [1,5]. Thus, this multisite study has two aims: (1) to develop an instrument derived from MP principles established in the literature, augmented with principles connected to the Chinese context; (2) to gather validity evidence in the Chinese context supporting the use of this inventory to assess physicians’ professional behaviors.

## Methods

### Study design and ethics

**Figure 1:**
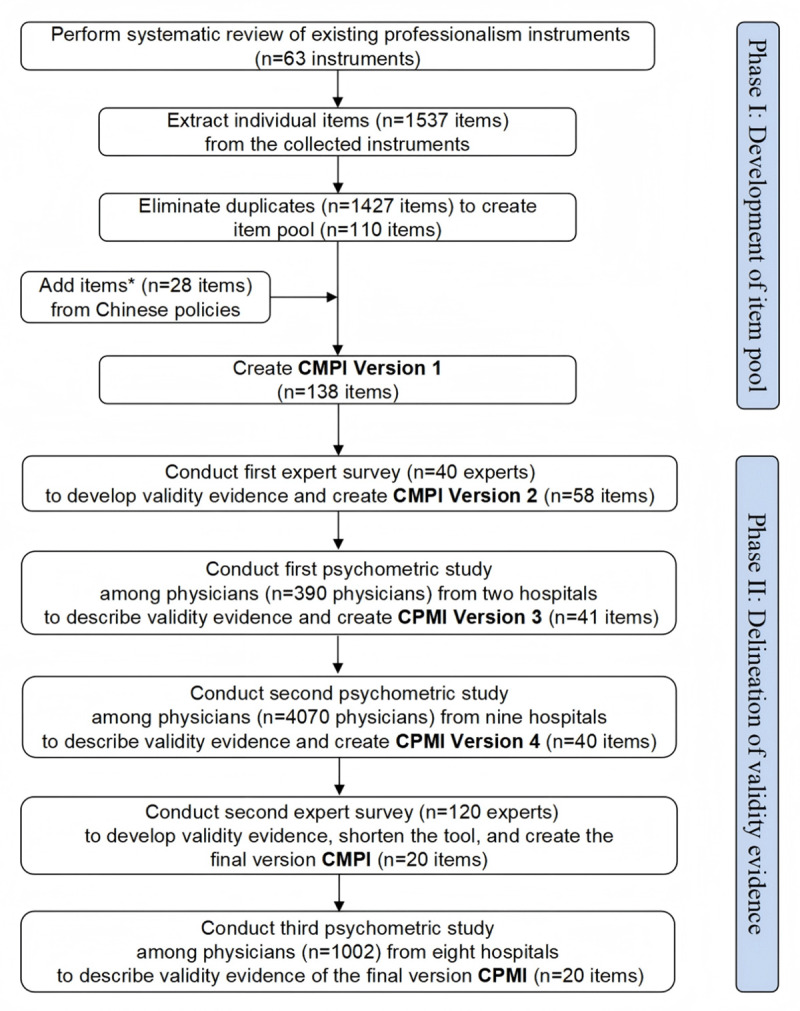
Flow diagram describing two phases of the development and validation processes of the China Medical Professional Inventory (CMPI). Black arrows indicate the steps to develop the CMPI and collect validity evidence. *The 28 added items are Chinese policy items not already found among the 110 items from systematic review and extraction.

We generated validity evidence aligned with Messick’s unified validity framework [31] and the current Standards for Educational and Psychological Testing [32]. We undertook a 2-phase process to develop the China Medical Professionalism Inventory (CMPI): Phase I to create the item pool; Phase II to delineate validity evidence, including content, internal structure, and relationships to other variables. Figure 1 summarizes the methods, participants, and items involved. Appendix 1 details processes, analyses, statistical terms, and standards. China Medical University’s Bioethics Advisory Commission (institutional review board), approved the protocol (ID: 2017075).

### Phase I: Development of item pool

We reviewed the literature reporting instruments that measure MP, combining key terms: *professionalism* AND *physicians* AND *instruments or assessing* AND *psychometric properties*.

We extracted items [13], and organized and eliminated duplicate items, using the framework commended by Lesser et al. [36]. We translated these items into Chinese. To develop evidence for cross-cultural validity, we followed Brislin’s modified model, an accepted process of forward-back translation and expert review [37,38]. To support this instrument’s representativeness with the Chinese healthcare context, we supplemented the item pool with MP-related Chinese government policies [39,40,41]. We converted items expressing attitudes or values into behavioral terms (e.g., “have commitment” was changed to “demonstrate responsibility for”), to create CMPI Version 1.

### Phase II: Delineation of validity evidence

We followed a standard practice [34,42,43] by inviting experts to review the CMPI Version 1 items. This process produced CMPI Version 2. We then conducted a series of psychometric studies to collect reliability and validity evidence as follows: the first psychometric study of CMPI Version 2 which led to CMPI Version 3; the second psychometric study of CMPI Version 3 which led to CMPI Version 4; an expert panel to shorten the survey and third psychometric analysis to describe the validity evidence of the final version CMPI (hereafter, “CMPI”) (Figure 1). Below we present participants, procedures, and analyses, organized by domain of validity evidence.

#### Participants and procedures

For the first expert survey, we invited 40 nationally prominent Chinese clinician-leaders using the following criteria: (1) Clinical experience and expertise in medical education, health system science, medical ethics, or MP; (2) Diversity of geographic location, including all seven administrative regions of China; and (3) Associate Professor or higher academic rank. Expert review was important for connecting to context [32,34] because the original pool derived from studies outside of China. Experts evaluated each item for fit (1 = Yes and 2 = No), relevance (1 = very low and 5 = very high), and importance (1 = very low and 5 = very high) for MP in China [42]. We phrased items to indicate behaviors; stems began, “The physician [action verb]…”. Following a standard approach for determining content-based evidence [31], experts could consider revisions or propose additional items. This process created CMPI Version 2.

The first psychometric study used CMPI Version 2 to survey 390 physicians from two tertiary teaching hospitals: China Medical University First Affiliated Hospital and Sheng-Jing Hospital. These hospitals are located in Shenyang, Liaoning Province and serve the general adult and pediatric population of this region of >43 million people [44]. We selected a stratified random sample of participants with equal proportions of physicians from major specialty disciplines (medicine, surgery, obstetrics and gynecology, pediatrics, radiology, and acute care). We used CMPI Version 2 items to calculate corrected item-total correlations (CITC), Cronbach’s Alpha if Item Deleted (CAID), exploratory factor analysis (EFA), and Cronbach’s alpha and produced CMPI Version 3.

The second psychometric study used CMPI Version 3 to survey 4,070 physicians from nine diverse hospitals (i.e., location, size, mission, ranking) in eight cities in Liaoning using a stratified cluster sampling method. This self-administered survey asked participants to indicate how often they demonstrate particular physician behaviors in practice, using a 5-point Likert-style scale (1 = not at all to 5 = very much). The sum of all items provided the total score, with higher scores indicating a higher frequency of self-reported MP behaviors. We used the approach of asking “how often” to attempt to mitigate some social desirability bias, a risk that may be equal or higher in Asian populations [45,46]. We used CMPI Version 3 items to calculate confirmatory factor analysis (CFA) and Cronbach’s alpha and produced CMPI Version 4.

We followed guidance to shorten the instrument using a second expert survey [47]. We provided CMPI Version 4 to 120 experts from 19 clinical disciplines in 17 medical universities across China. We considered “experts” as educators eligible to consult with China’s National Medical Examination Center (NMEC)—the organization overseeing China’s medical licensing exam. The NMEC chooses consultants for their expertise in clinical medicine, medical education, and assessment. We asked the experts to select the five most important MP items for each factor. The expert survey produced the final 20-item CMPI. We sent the CMPI to 1,002 physicians selected by stratified random sampling, with equal proportions from eight tertiary hospitals in Liaoning. We calculated CFA and Cronbach’s alpha to produce validity evidence for CMPI. For each psychometric study, we collected participants’ demographic information.

#### Analysis

We collected evidence based on content, internal structure, and relationship to other variables.

#### Content evidence

Content evidence for validity included Phase I’s development of the item pool (i.e., literature review, item synthesis) and Phase II’s two expert surveys.

During the literature review, we evaluated the methodological quality of MP instruments using the COnsensus-based Standards for the selection of health status Measurement INstrument (COSMIN) checklist [13,48]. COSMIN is “a widely accepted framework developed for systematically evaluating the methodological quality of studies” [15 (p. 2)], including assessments of physicians [49,50]. We only included instruments that met COSMIN standards.

For the first expert survey, we followed the accepted standards for defining consensus [42]: We retained items when >90% of the experts determined the item fit the Chinese context and mean scores for both relevance and importance were >4. The second expert survey aimed to shorten the tool and maintain reliability. To support content validity, we used a “content evaluation panel” in which experts’ evaluations and quantitative metrics systematically identify essential items [51]. We decided in advance to maintain the top 50% of experts’ preferred items to prevent the experts leaving the survey longer than necessary (i.e., less feasible without improved reliability). To support evidence based on content, we ensured that experts involved in Phase I and Phase II surveys, like the population surveyed in the psychometric studies, were practicing clinicians from a diverse array of specialties.

#### Internal structure

For the first psychometric study, we conducted item analysis using CITC and CAID [52,53]. We performed Bartlett’s Test of Sphericity, the Kaiser–Meyer–Olkin (KMO) test, and EFA to describe the underlying structure of the instrument [32,33]. We calculated Cronbach’s alpha to determine internal-consistency reliability for the total scale and each factor from EFA [32,52,54]. For the second psychometric study, we examined evidence based on internal structure of the scale using CFA to evaluate the hypothesized factorial structure [33]. Using the EFA results from the first psychometric study, we fit the data into a 4-factor model by deleting items with a factor loading of <0.45. We used five standard tests to assess model fit (Table 3 footnote) [55]. For the third psychometric study, we used CFA and Pearson correlation coefficients to provide evidence based on internal structure and Cronbach’s alpha to assess internal-consistency reliability of the shortened, 20-item CMPI [43].

#### Relationships to other variables

During the second psychometric study, we investigated convergent evidence using Pearson correlation coefficients [43]. Using the Chinese version of the Penn State College of Medicine Professionalism Questionnaire (PSPQ) [56], a recognized MP instrument developed in the West, we expected a moderate correlation with the CMPI. Because prior studies report that physicians’ burnout level *negatively* correlates with MP [57], we administered the short version of the Maslach Burnout Inventory (MBI) [58], expecting an association (negatively) with this scale.

For statistical analyses, we used IBM SPSS (IBM Corp. Released 2011, Version 20.0. Armonk, NY: IBM Corp. Chicago, IL, USA) and Amos Version 21.0 (Chicago: IBM SPSS. Released 2012) for CFA. We set statistical significance at *P* < 0.05 (two-tailed tests).

## Results

We describe validity evidence for content, internal structure, and relationships to other variables and identify the phase in which data were collected.

### Validity evidence for content

In Phase I, we extracted all items from 63 tools, eliminated duplicates, and added Chinese policy items to produce CMPI Version 1 (Figure 1). In Phase II, the first expert survey included 34 (85%) completed questionnaires from examination experts. Experts represented 27 medical schools throughout China; each was ranked “first class” on the Chinese government’s official listing. We eliminated 82 items (71 from our original literature review and 11 from Chinese policies) the experts determined were less related or less important. The resulting 56-item pool included 17 items sourced from policy documents. The experts recommended two additional items: “respects patient autonomy and their informed decisions” and “shares experience, skills, and knowledge with junior colleagues,” resulting in a 58-item CMPI Version 2 (Table 1). In Phase II, the second expert survey included 76 clinicians (response rate, 63%) who ranked the importance of each item. For each factor confirmed by CFA, we retained the top 50% of items to generate the 20-item CMPI (Table 1 and Appendix 2).

**Table 1:** Items Included in Three Versions of the China Medical Professionalism Inventory (CMPI).


FACTOR	ITEM NO.^$^	ITEM	CMPI VERSION 2 (58 ITEMS)	CMPI VERSION 4 (40 ITEMS)	CMPI (FINAL) (20 ITEMS)

Respect, Compassion, and Communication	1	Attends to psychological and emotional factors and socio-psychological factors related to the patient’s health.	√		

	2	Talks about preventative care (e.g., quitting smoking, weight control, sleeping, alcohol, exercise, etc.).	√		

	3	Presents professional opinion to the patient in a way the patient can understand.	√	√	√

	4	Takes the time and effort necessary to explain information to patients.	√		

	5	Helps the patient with their fears and worries.	√		

	6	Explains to the patient what they need to know about their problems, how and why they occurred, and what to expect next.	√	√	√

	7	Takes the patient’s embarrassment, shyness, and reluctance into account and provides timely emotional support when necessary.	√	√	

	8	Demonstrates respect for patient autonomy by ensuring patients understand their situation and make informed clinical decisions. [added by expert review*]	√	√	√

	9	Does not use degrading or mocking words when discussing a patient with coworkers.	√		

	10	Does not discriminate against or refuse to treat a patient due to gender, race, religion, nationality, family background, sexual orientation, or economic status. *[Chinese policy^]*	√	√	

	11	Greets patients warmly; calling them by the names they prefer; is friendly, never crabby or rude.	√	√	

	12	Deals appropriately with a patient who is emotionally out of control.	√		

	13	Follows the regulations and procedures for declaration of a patient’s death and takes care of the family’s emotions to give proper comfort. *[Chinese policy^]*	√	√	

	14	Discusses options with patients, asks their opinions, offers choices, and lets them decide what to do before making decisions.	√	√	√

	15	Does not treat the patient’s informed consent for surgery, tests, or treatment as a means to escape from responsibility. *[Chinese policy^]*	√		

	16	Follows the patient’s preference to accept or refuse any clinical treatment.	√	√	√

	17	Places their patient’s convenience before their own when arranging the tests and treatment.	√		

	18	Follows the patient’s decisions before loss of cognitive capacity and protects their rights and interests by means of a will or alternative consent by a close relative. *[Chinese policy^]*	√		

	19	Maintains a positive rapport with the whole healthcare team and provides emotional support for colleagues.	√	√	√

	20	Works collaboratively across disciplines to complete medical responsibilities.	√	√	

	21	Demonstrates respect for clinical assistants, such as nurses and other staff.	√	√	

	22	Demonstrates trust in the professional knowledge and skills of coworkers.	√	√	

	23	Resolves interdisciplinary conflicts in a collegial and respectful manner.	√	√	√

Integrity	24	Avoids discussing and revealing confidential patient information in public.	√	√	√

	25	Maintains patient/physician relationships that do not exploit personal financial gain, privacy, or sexual advantages.	√	√	√

	26	Actively reports any personal medical or research errors.	√		

	27	Acts to minimize the possibility of treatment failure and medical error.	√		

	28	Takes responsibility for their own clinical decisions and medical practices (i.e., they do not seek to evade responsibility).	√	√	

	29	Takes responsibility even in the face of difficulties.	√		

	30	Does not allow any possible personal benefit to impact on professional behavioral and decision making. *[Chinese policy^]*	√		

	31	Avoids conducting non-scientific or unethical research supported by commercial sponsorship. *[Chinese policy^]*	√	√	√

	32	Does not sell any medical products or prescribe drugs for personal benefit. *[Chinese policy^]*	√	√	

	33	Does not participate in commercially sponsored banquets, tourism, training, or other activities that may lead to medical bias. *[Chinese policy^]*	√	√	

	34	Does not attract patients through misleading advertising. *[Chinese policy^]*	√	√	

	35	Participates in peer evaluations of the quality of care provided by colleagues objectively.	√	√	

	36	Reports colleagues’ misconduct or medical error to a hospital or a professional organization, and does not shield peers. *[Chinese policy^]*	√		

	37	Explains treatment risks to patients fully and does not give patients false hope.	√	√	√

	38	Provides appropriate and clear information to colleagues for follow-up patient care.	√	√	√

Excellence	39	Follows scientific standards and bases decisions on scientific evidence and experience.	√	√	

	40	Applies new clinical practice guidelines into patient care actively and independently.	√	√	√

	41	Acknowledges the meaning and relative value of scientific evidence in decision-making.	√	√	√

	42	Uses practical experience as a basis for critical self-reflection.	√	√	

	43	Consults other medical colleagues to manage a situation that is beyond one’s ability.	√	√	√

	44	Seeks additional learning opportunities to acquire new knowledge and skills to remain current in their profession.	√		

	45	Engages in continuous professional development (CPD).	√	√	

	46	Commits to keep up with current academic literature and participate in peer discussions in their field. *[Chinese policy^]*	√		

	47	Promotes the welfare and career of junior faculty.	√	√	

	48	Shares experience, skills, and knowledge with junior colleagues. [added by expert review*]	√	√	√

Responsibility	49	Pays attention to the risk factors that may threaten the safety of the patient by actively providing early warning and improvement suggestions to the relevant authority. *[Chinese policy^]*	√	√	√

	50	Does not provide unnecessary or excessive testing or medical treatment. *[Chinese policy^]*	√	√	√

	51	Does not change care practice due to the social status and economic situation of the patient.	√		

	52	Advocates for public health and transfers knowledge of public health to patients. *[Chinese policy^]*	√	√	

	53	Ensures that the patient understands the content and meaning of the informed consent correctly and fully.	√	√	√

	54	Does not participate in or support behavior or academic activities that go against humanism. *[Chinese policy^]*	√		

	55	Distinguishes between accepted treatment and experimental activities and abides by ethical standards.	√	√	√

	56	Does not harm the patient or put the patient at unnecessary risk by using medical knowledge and skills that the doctors know to not be in the best interest of the patient. *[Chinese policy^]*	√	√	

	57	Collaborates with peers to avoid unnecessary tests and optimizes the use of medical resources.	√	√	

	58	Chooses appropriate medical treatment for a patient with financial constraints and helps them in finding other means of assistance. *[Chinese policy^]*	√	√	


^$^ Item numbers correspond to the 58-item CMPI Version 2, used for the first of three psychometric studies. ^Added from Chinese policies about medical professionalism during Phase I. *Added by the first expert survey in Phase II.

Evidence for content was supported by ensuring that all survey participants were clinicians. The first survey’s 34 experts, the second survey’s (different) 76 experts, and survey respondents in the three psychometric studies (Table 2) all had clinical medical training and practice experience, representing an array of disciplines.

**Table 2:** Characteristics of physicians from psychometric studies of three versions of the China Medical Professionalism Inventory (CMPI), 2017–2018.


CHARACTERISTIC	GROUP	1^ST^ PSYCHOMETRIC STUDY, n (% OF 360 PHYSICIANS)^c^	2^ND^ PSYCHOMETRIC STUDY, n (% OF 3653 PHYSICIANS)^d^	3^RD^ PSYCHOMETRIC STUDY, n (% OF 955 PHYSICIANS)^e^

Sex	Male	183 (50.8)	1777 (48.6)	472 (49.4)

	Female	176 (48.9)	1870 (51.2)	480 (50.2)

Age	≤30	84 (23.4)	927 (25.4)	176 (18.4)

	31–40	181 (50.4)	1420 (38.9)	497 (52.0)

	41–50	69 (19.2)	789 (21.6)	200 (21.0)

	51–60	24 (6.7)	433 (11.9)	66(7.0)

	≥61	1 (0.3)	16 (0.5)	5 (0.5)

Educational level^a^	Doctorate degree	179 (47.2)	750 (20.5)	214 (22.4)

	Master’s degree	175 (48.6)	1690 (46.3)	490 (51.3)

	Bachelor’s degree	11 (3.1)	1152 (31.5)	225 (23.6)

	Other degree	1 (0.3)	33 (0.9)	11 (1.2)

Specialization	Internal medicine	139 (38.6)	1543 (42.2)	392 (41.4)

	Surgery	145 (40.3)	1207 (33.0)	367 (38.4)

	Obstetrics and Gynecology	24 (6.7)	203 (5.6)	61 (6.4)

	Pediatrics	11 (3.1)	113 (3.1)	42 (4.4)

	Intensive care and other^b^	39 (10.8)	378 (10.1)	74 (7.8)


^a^ In the Chinese medical education system, medical school begins at the undergraduate level. The Chinese Bachelor of Medicine, Bachelor of Surgery (MBBS) degree is regarded as the equivalent of the Doctor of Medicine (MD) degree in the United States. The master’s degree is in addition to MBBS, and a PhD is in addition to MBBS or a master’s degree. The “other degree” refers to qualifications below MBBS, such as associate degrees or technical secondary school diplomas. ^b^ The category “other” includes Dermatology, Otorhinolaryngology, Intensive Care, etc. ^c^ The 1^st^ psychometric study used CMPI Version 2, which produced CMPI Version 3. ^d^ The 2^nd^ psychometric study used CMPI Version 3, which produced CMPI Version 4. ^e^ The 3^rd^ psychometric study provided validity evidence for the final, 20-item CMPI.

### Validity evidence for internal structure

In Phase II, 360 physicians (response rate, 92.3%) returned the first psychometric study, and 3653 physicians (response rate, 89.8%) returned the second psychometric study. For the third psychometric study, 955 clinicians (response rate, 95.3%) completed the CMPI. Table 2 presents the socio-demographic characteristics of these physician respondents.

For the first psychometric study, we deleted 14 items that did not meet CITC and CAID standards [52,53], removed 19 surveys (5%) with missing data, and analyzed 341 responses for EFA. Using the remaining 44 items for EFA, both Bartlett’s Test of Sphericity (χ^2^ = 16,281, *P* < 0.001) and the KMO test (0.98) indicated that the data were factorable. EFA determined four factors (eigenvalues >1.0). Three items did not meet the predetermined standard (factor loading ≥0.45); thus, CMPI Version 3 retained 41 items for the second psychometric study. We labeled the four factors according to the common theme shared among the items: “Respect, Compassion, and Communication”; “Integrity”; “Excellence”; and “Responsibility.” The four factors account for a total of 66.65% of the scale variance, an acceptable standard [43].

For the second psychometric study, we removed 149 surveys (4%) because of missing data and analyzed 3504 responses for CFA. Additionally, one item was removed: item 26 (“Actively reports any personal medical or research errors”). Following a standard process [33], we deleted this item because it loaded strongly on *two* factors on EFA: “Integrity” (0.45) and “Excellence” (0.46). The subsequent CFA provided a satisfactory fit to the 4-factor model (Table 3). This process yielded the 40-item CMPI Version 4 (Table 1).

**Table 3:** Measures of fit for two rounds of confirmatory factor analysis (CFA).


MODEL	χ^2^, *df, P*-VALUE, NORMED χ^2^^a^	CFI^b^	TLI^c^	RMSEA (95% CI)^d^	SRMR^e^

Phase II:Second psychometric study CFA	χ^2^ = 3305.3, *df* = 735, *P* < .0001, Normed χ^2^ = 4.50	0.93	0.91	0.059 (0.053–0.066)	0.036

Phase II: Third psychometric study CFA	χ^2^ = 660.5, *df* = 166, *P* < .0001, Normed χ^2^ = 3.98	0.97	0.97	0.060 (0.054–0.066)	0.009


Abbreviations: χ^2^, chi-square test; *df*, degrees of freedom; CFI, comparative fit index; TLI, Tucker-Lewis index; RMSEA root mean square error of approximation; CI, confidence interval; SRMR, standardized root mean square residual. ^a^ For model fit, when calculating χ^2^ for CFA, a *P*-value >0.05 is considered “significant” suggesting the proposed model represents the data; this standard, however, is affected by sample size. With samples sizes >200 (e.g., as in our study: we sampled 3504 and 803 in our two CFAs), the *P*-value will nearly always be >0.05 which undermines the utility of using χ^2^ for CFA. The recommended statistical approach is to use “normed” χ^2^ wherein *P* < 0.05 is acceptable. ^b^ CFI calculations, a measure of model fit, estimate the proportion of sample data the proposed model explains. CFI measurements adjust for sample size issues that exist when calculating model fit by the chi-square test. CFI values range from 0 to 1; values above 0.90 are generally considered acceptable. CFI should be used in the context of other results and not as a single strict cut-off. ^c^ TLI is one of several calculations to determine “model fit.” Model fit is an overall determination of the degree to which the data confirm the proposed model (in our study, the model is shown in Figure 2). TLI values of >0.80 are usually considered acceptable. ^d^ RMSEA is one of several calculations to determine “model fit.” RMSEA calculations take into account degrees of freedom in the covariance matrices. RMSEA results represent standardized differences between proposed model and predicted models. RMSEA values <0.08 are considered an acceptable fit of the data to the proposed model. ^e^ SRMR is one of several calculations to determine “model fit.” The SRMR takes into account the standardized differences between proposed model and predicted models. The SRMR predictions of goodness of fit may be less affected by sample size. SRMR values <0.08 are considered an acceptable fit of the data to the proposed model.

**Figure 2:**
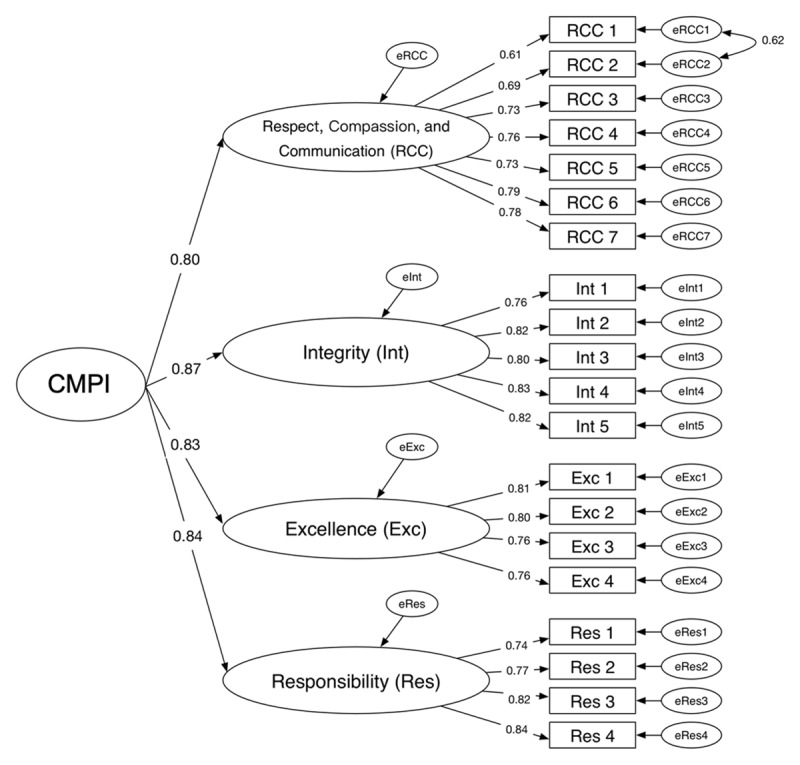
Confirmatory factor analysis (CFA) of the final, 20-item China Medical Professionalism Inventory (CMPI). Path coefficients appear as numeric values on the arrows connecting CMPI to its subscales. Each coefficient indicates the relationship between CMPI and its factors. Item factor loading appears as numeric values on the arrows connecting factors to corresponding individual items, which are all above 0.5. We adjusted the model based on the modification indices that indicated the correlation between error terms for two items on the “Respect and Communication” (RCC) factor (RCC item 1 and RCC item 2). *Abbreviations:* RCC indicates respect, compassion, and communication; Int, integrity; Exc, excellence; and Res, responsibility.

For the third psychometric study, we removed 152 surveys (15.9%) because of missing data and analyzed 803 responses for CFA. After we adjusted for correlated item pairs, the subsequent CFA indicated that all 20 items loaded significantly (*P* < 0.01) on their respective factors (Figure 2). The CFA indicated a satisfactory fit for the 4-factor model (Table 3).

### Additional internal structure evidence

For the first and second psychometric studies, Cronbach’s alpha coefficients for each factor were >0.9. The results of the second study produced CMPI Version 4 (respondents’ mean score = 182.5, SD = 20.46; median = 188; range of scores = 54–200). The third psychometric study provided additional evidence of the final CMPI’s internal structure (Appendix 3). The internal-consistency reliability of the total scale and four factors are all in a satisfactory level (Cronbach’s alpha >0.7). The final CMPI respondents’ mean score was 88.3 with SD = 20.47 (median = 89; range of scores 60–100).

### Validity evidence for Relationships to other variables

Our results indicated that the CMPI correlated positively with the Chinese version of the PSPQ (Pearson *r* = 0.77, *P* < .01) and correlated negatively with the short version MBI (Pearson *r* = –.22, *P* < .01).

### Chinese-derived items

After expert surveys and psychometric studies, the CMPI retained items derived from three sources: the initial item pool, Chinese healthcare policies not found in the initial item pool, and additional items provided by Chinese experts (Appendix 4). Items originating from policies and expert supplementation accounted for 25.0% of the total of the CMPI. The two items added by experts, “Demonstrates respect for patient autonomy…” and “Shares experience…with junior colleagues” (Table 1: items #8 and #48), were retained throughout the process.

## Discussion

We developed the China Medical Professionalism Inventory to assess MP of Chinese physicians and collected validity evidence from two expert surveys and three psychometric studies. Our analysis suggests that the process of developing the CMPI in the Chinese context included supportive evidence based on content, internal structure, and relationships to other variables. The EFA and CFA determined that the CMPI has four factors (“Respect, Compassion, and Communication,” “Integrity,” “Excellence,” and “Responsibility”).

The CMPI differs from prior MP studies in Eastern contexts in three ways: (1) The processes to determine validation evidence for the CMPI included large surveys in the Chinese context that underpinned the psychometric analysis; (2) Chinese experts had the opportunity to include or remove Western-derived items; (3) The process offered opportunities to incorporate items reflecting traditional Chinese cultural values and current policies. To our knowledge, the CMPI is the first behaviorally referenced MP scale incorporating Chinese health system policies and surveying Chinese experts for scale development. Below, we summarize our processes of collecting validity evidence and comment about future research. We reflect on the CMPI’s Chinese-derived elements, the Western-derived Physician Charter, and the Chinese context.

### Validity Evidence

We organize our results and validity evidence following Messick and the Standards frameworks: content, internal structure, and relationship to other variables [31,32]. We developed evidence based on content by surveying two distinct groups of national experts from across China to assess each item’s fit, importance, and contextual relevance. We administered three psychometric studies to physicians to provide evidence based on internal structure of the 4-factor model. To assess relationships to other variables, we determined convergent evidence. The CMPI showed a strong positive correlation with the PSPQ that assesses MP attitudes and a negative correlation, albeit weak, with the MBI that assesses physician burnout.

The CMPI has 20-items, and the tool’s Cronbach’s alpha suggests room to reduce the number of items further [54]. For example, the CMPI uses *self-reports* of behaviors. To assess professional behaviors of *other* physicians (not oneself), future research could remove items that are more cognitive and not clearly observable (i.e., CMPI items #14 and #20) and collect validity evidence from that new CMPI version [15]. The process of reducing the items could also provide information on those new tools’ relative test-retest and interrater reliability.

The CMPI supports *the beginning* of a process of MP assessment in China, and potentially beyond. Such tools could even support improved care of patients [14]. Subsequent iterations of the CMPI will require new validation evidence in the context of its use, judgment about scoring, generalization, extrapolation, and implication. Future research should include consequential validity, response process [32] and “qualitative and subjective data” from “multiple assessment data points” (p. 561) [34]. Ultimately, tool development should involve “the accumulation of evidence across time, settings and samples to build a scientifically sound validity argument….an ongoing process” (p. 465) [59].

### East Meets West

Just as Western tools are used in the East, one could imagine that instruments developed in Eastern contexts, with validation evidence, could inform Western conceptualizations of MP. How much convergence and divergence occurs when the CMPI, derived in one Eastern context, “meets West”?

The CMPI appears to have integrated elements of traditional Chinese culture and contemporary policy requirements with Western conceptualizations of MP. We developed the item pool from Western MP assessment tools, Chinese policies, and Chinese experts. Appendix 4 documents the CMPI’s Chinese-derived items—those retained and those lost in the development process. Appendix 5 broadens our lens and compares multiple Western and Eastern tools.

As one example, we compare the CMPI to a widely applied Western framework, the Physician’s Charter [60]. Two observations stand out: (1) As with prior studies comparing Chinese and Western MP frameworks, many items in the CMPI overlap the Charter while some appear culturally particular [11,16,17,18], and (2) some items appear to reflect differing current emphases in Chinese and Western contexts.

Regarding areas of overlap, 15 of the CMPI’s 20 items (75%) originate from the initial item pool before the Chinese policy items were added. Of the five Chinese policy items, four map to one or more of the core principles of the Charter: autonomy, welfare, and social justice [60]. For example, “Avoids conducting non-scientific or unethical research…” and “Does not provide unnecessary or excessive testing…” might be considered within the Charter’s “Principle of Patient Autonomy” and “Principle of Primacy of Patient Welfare” [60]. One CMPI item “Shares experience…with junior colleagues” is hard to map to the Charter’s principles.

Regarding areas of differing emphasis, we note the earlier CMPI versions retained fewer items about care equity. Another core element of the Charter, care access [60], was omitted entirely in the CMPI. We speculate that surveyed physicians and experts did not retain these items because in China, these behaviors are viewed as culturally foundational—so basic that they would not require MP assessment. In China, “social justice” has deep Confucian roots as a longstanding, expected, and enacted value across society. Since the pre-Qin Dynasty (c.261 BCE) [61], Confucianism has “emphasized human equality overall” and a communal Confucian ethic of “justice” (义, yi) [p. 199, 62]. These principles underpin the requirement of China’s universal health care system to provide access and just distribution of healthcare to all people—rural and urban, wealthy and less resourced, etc. [63,64,65]. The system prioritizes access over the Western notion of individual experience of care [66,67]. The health system, like other sectors of Chinese society, animates Confucianism and supports China’s collectivist culture; we suspect that the transcendent, commonplace nature of these values obviated the impulse for respondents and experts to maintain those items.

As well, the contextually derived CMPI differs from the Charter where the CMPI added items connected to China’s recent 10-year national healthcare reform [68,69,70]. This reform emphasizes conflicts of interest, new payment policies, financial integrity, and ending the relationship between physicians’ income and drug dispensing and laboratory examinations [68,70]. The CMPI also includes a high proportion of items related to communication, shared decision-making, and respecting patient autonomy, domains related to new conceptualizations of the doctor-patient relationship in China [69,71,72] and the East more broadly. This approach to MP reflects a change from earlier Chinese medical practice which was hierarchical and paternalistic, whereby patients (and junior colleagues) viewed doctors as powerful authority figures who direct patients’ care [69,71,72].

We observe another difference in emphasis between the CMPI and the Charter. In the 58-item CMPI Version 2 (Table 1), item #36 states physicians should “report colleagues’ misconduct…” In China, peer reporting is akin to “whistle-blowing”—a socially unacceptable behavior due to Confucian emphases on social conformity, harmony, and a commitment to trust and loyalty in relationships [73,74]. In contrast, the Charter’s “Commitment to professional responsibilities” calls explicitly for “remediation and discipline of members who have failed to meet professional standards” and “individual and collective obligations to…accepting external scrutiny of all aspects of their professional performance” [60].

Finally, we believe that the development of the CMPI aligns with the Chinese traditional philosophy of “inclusiveness” and “harmony”—to learn and absorb ideas outside China while preserving core values and cultural foundations. To support and advance MP, this approach encourages a commitment to exploration and openness, grounded in respect for multiculturalism.

### Limitations

This study has limitations. Our original and repeat literature searches could have missed tools and items. We conducted psychometric studies in Liaoning Province, and regional factors could influence results. Our sample demographics compared with China’s overall physician demographics [44], but unmeasured factors could influence results. We surveyed urban, tertiary hospital physicians and experts, missing views of rural providers. We note Chinese MP challenges are most reported among urban, tertiary-trained physicians [71,72]. Our multidisciplinary experts were diverse geographically and diversely experienced, but we did not collect their demographic data. Although all participants were practicing clinicians, we did not undertake cognitive interviewing to produce evidence for response process.

Regarding evidence based on internal structure, our three rounds of surveys relied on self-reports, which has limitations [75] and risks social desirability bias [45]. We attempted to reduce this bias by wording questions for physicians as frequencies (i.e., how often), wording questions for expert reviewers indirectly [46] (“what *should* a physician demonstrate”), and informing survey participants and experts that surveys were anonymous, and participants were not individually judged or assessed. We did not develop consequential validity evidence.

## Conclusion

To better characterize, support, and foster MP in China, leaders within and outside of medicine will require trustworthy instruments. Such tools should be culturally aligned with ongoing demonstration of validity evidence within the context of their use. We created the CMPI as a behaviorally referenced tool to meet these requirements. Recognizing the practice of applying Western MP tools in the East, we hope the contextually derived CMPI may support MP in China, and potentially further our understanding of professionalism more broadly as “East meets West.”

## Previous presentations

An early presentation as “research-in-progress” was presented internally within China Medical University, Li Honghe’s institution, in their Institute of Health Professions Education Assessment and Reform in 2021.

## Additional Files

The additional files for this article can be found as follows:

10.5334/pme.1682.s1 Appendix 1. Details of processes, analyses, statistical terms, and standards.

10.5334/pme.1682.s2 Appendix 2. China Medical Professionalism Inventory (CMPI) in English and Chinese, 20 items.

10.5334/pme.1682.s3 Appendix 3. Interfactor correlations and Cronbach’s alpha coefficients of the 20-item China Medical Professionalism Inventory (CMPI).

10.5334/pme.1682.s4 Appendix 4. Summary of Items Included and Excluded from Chinese Policies and Experts.

10.5334/pme.1682.s5 Appendix 5. Characteristics of Existing Instruments.
