# Supplementary material for: East Meets West: A Multisite Validity Study of the China Medical Professionalism Inventory
Source: Perspect Med Educ. 2025 Sep 25;14(1):603–18. doi: 10.5334/pme.1682 (PMC12466328; doi:10.5334/pme.1682)
Supplement: Appendix 1. — Details of processes, analyses, statistical terms, and standards. [file pme-14-1-1682-s1.pdf]

Appendix 1: Details of processes, analyses, statistical terms, and standards

Full description of the Phase I process

Search strategy
We reviewed the literature reporting instruments that measure medical professionalism (MP), combining key terms: professionalism AND physicians AND instruments or assessing AND psychometric properties by Applying Terwee's search construct[1]. We systematically searched the English language literature using PubMed, Web of Science, and PsycINFO databases to identify studies and instruments assessing MP between January 1, 1990 and December 31, 2015[2]. At that time, our search of the Chinese-language literature revealed no Chinese MP tools with validity evidence. Repeat searches through April 2025 did not find new tools or items that met our criteria.

Item extraction
To create the initial item pool, three researchers (H.L., X.S., X.Y.) extracted items from existing MP instruments identified by the systematic review[2]. We extracted items,[2] and organized and eliminated duplicate items, using the framework commended by Lesser et al.[3]

Translation process
We translated these items into Chinese. To develop evidence for cross-cultural validity, we followed Brislin's modified model, an accepted process of forward-back translation and expert review for "ensuring cross-cultural congruency or equivalency between the two languages" and minimizing bias in cross-cultural adaptation (p. 77)[4]. Our bicultural, bilingual researchers who are Chinese (H.L. and X.S.) and Chinese-Canadian (N.J.), created the item pool through several rounds of blinded forward and back translation. Two pairs of researchers (H.L. with D.M. and H.L. with D.A.H.) separately ensured the final English and Chinese translations met standards for content, semantic, technical, criterion, and conceptual equivalence[5].

Description of analyses, statistical terms, and standards

Internal consistency analysis
We used Cronbach's alpha to assess internal consistency, which is the degree of the interrelatedness among the items[6]. Following a widely used standard in the literature, we considered alpha values $\geqq 0.7$ to be acceptable[7,8].

Item analysis
We calculated corrected item-total correlations (CITC) to determine associations of each item with the total scale score (total score from all other items); CITC >0.5 considered is acceptable[7,9]. We determined Cronbach's alpha if item deleted (CAID) to delete items with a CAID higher than the total scale Cronbach's alpha. When an item has a CAID higher than the total scale alpha, removing the item would improve internal consistency of the total scale.

Exploratory Factor Analysis (EFA)
We performed EFA to describe the underlying structure of the instrument[6,10] using Principal Component Analysis with Varimax rotation to extract the factors. We evaluated assumptions

regarding matrix identity and sampling adequacy using Bartlett's Test of Sphericity and the Kaiser–Meyer–Olkin (KMO) test. These indicators are employed to evaluate the adequacy of the data for factor analysis and to ascertain the appropriateness of the variable set for meaningful factor extraction. Eigenvalues are a measure of the amount of variance in the original variables that is explained by each factor. A higher eigenvalue indicates that a factor explains a larger proportion of the variance in the data. To be included in the inventory, each factor needed to have an eigenvalue >1 and at least three items. Factor loadings are essentially standardized regression coefficients, indicating how much an item is related to a factor. Each item needed to have a factor loading ≧0.45.

Confirmatory Factor Analysis (CFA)[11]
(Table 3)
We calculated five standard tests for CFA to provide evidence of internal structure:
A) χ^2 (chi-square) test. For model fit, when calculating χ^2 for CFA, a *P*-value >0.05 is considered "significant" suggesting the proposed model represents the data; this standard, however, is affected by sample size. With samples sizes >200 (e.g., as in our study: we sampled 3504 and 803 in our two CFAs), the *P*-value will nearly always be >0.05 which undermines the utility of using χ^2 for CFA. The recommended statistical approach is to use "normed" χ^2 wherein *P*<0.05 is acceptable.
B) CFI, comparative fit index. CFI calculations, a measure of model fit, estimate the proportion of sample data the proposed model explains. CFI measurements adjust for sample size issues that exist when calculating model fit by the chi-square test. CFI values range from 0 to 1; values above 0.90 are generally considered acceptable. CFI should be used in the context of other results and not as a single strict cut off.
C) TLI, Tucker-Lewis index. TLI is one of several calculations to determine "model fit." Model fit is an overall determination of the degree to which the data confirm the proposed model. TLI values of >0.80 are usually considered acceptable.
D) RMSEA, root mean square error of approximation. RMSEA is one of several calculations to determine "model fit." RMSEA calculations take into account degrees of freedom in the covariance matrices. RMSEA results represent standardized differences between proposed model and predicted models. RMSEA values <0.08 are considered an acceptable fit of the data to the proposed model.
E) SRMR, standardized root mean square residual. SRMR is one of several calculations to determine "model fit." The SRMR takes into account the standardized differences between proposed model and predicted models. The SRMR predictions of goodness of fit may be less affected by sample size. SRMR values <0.08 are considered an acceptable fit of the data to the proposed model.

Correlation analysis
We investigated convergent evidence using Pearson correlation coefficients[12]. We used this calculation to provide an estimate of the relationship between the CMPI and scales with constructs we deemed to be related. We investigated the correlation between the CMPI scores and the scores the Chinese version of the Penn State College of Medicine Professionalism Questionnaire (PSPQ)[13] and the short version of the Maslach Burnout Inventory (MBI)[14]. We predicted a positive correlation with the PSPQ and a negative correlation with the MBI. Higher Pearson correlation coefficients suggest a stronger relationship (positive or negative) between the

two compared scales and thereby offer evidence of convergent validity. Pearson coefficients are presented as "*r*" ranging from -1 to 1. A positive "*r*" indicates a positive correlation and negative "*r*" indicates negative correlation. The statistical significance of the "*r*" result is presented with standard *P* value which researchers should define at the outset. In this study, we set statistical significance at $P < 0.05$ (two-tailed tests).

References

1. Terwee CB, Jansma EP, Riphagen II, de Vet HC. Development of a methodological PubMed search filter for finding studies on measurement properties of measurement instruments. Qual Life Res. 2009;18(8):1115-1123. DOI:10.1007/s11136-009-9528-5
2. Li H, Ding N, Zhang Y, Liu Y, Wen D. Assessing medical professionalism: A systematic review of instruments and their measurement properties. PLoS One. 2017;12:e0177321. DOI: 10.1371/journal.pone.0177321
3. Lesser CS, Lucey CR, Egener B, Braddock CH 3rd, Linas SL, Levinson W. A behavioral and systems view of professionalism. JAMA. 2010;304(24):2732-2737. DOI:10.1001/jama.2010.1864
4. Brislin RW. Back-translation for cross-cultural research. Journal of Cross-Cultural Psychology. 1970;1(3):187–216.
5. Lee CC, Li D, Arai S, Puntillo K. Ensuring cross-cultural equivalence in translation of research consents and clinical documents: a systematic process for translating English to Chinese. J Transcult Nurs. 2009;20(1):77-82. DOI:10.1177/1043659608325852.
6. Standards for educational and psychological testing. Washington, USA: American Educational Research Association, 2014
7. Streiner DL, Norman GR, Cairney J. Health measurement scales: a practical guide to their development and use. Oxford university press; 2024 Feb 8.
8. Nunnally, JC. (1978). Psychometric theory (2nd ed.). New York: McGraw-Hill.
9. Ferketich S. Focus on psychometrics. Aspects of item analysis. Res Nurs Health. 1991;14(2):165-168. DOI:10.1002/nur.4770140211
10. Cook DA, Beckman TJ. Current concepts in validity and reliability for psychometric instruments: theory and application. Am J Med. 2006;119:166.e7-16. DOI: 10.1016/j.amjmed.2005.10.036.
11. Hu LT, Bentler PM. Cutoff criteria for fit indexes in covariance structure analysis: Conventional criteria versus new alternatives. Structural equation modeling: a multidisciplinary journal. 1999;6(1):1-55. DOI: 10.1080/10705519909540118.
12. Boateng GO, Neilands TB, Frongillo EA, Melgar-Quiñonez HR, Young SL. Best practices for developing and validating scales for health, social, and behavioral research: A primer. Front Public Health. 2018;6:149. DOI: 10.3389/fpubh.2018.00149
13. Song W, Shi L, Li H, Wen D. The introduction of the Penn State College of Medicine Professionalism Questionnaire and the evaluation on its validity and reliability. Chinese Journal of Medical Education. 2019;39:868 ‒ 871. *(in Chinese)*
14. West CP, Dyrbye LN, Sloan JA, Shanafelt TD. Single item measures of emotional exhaustion and depersonalization are useful for assessing burnout in medical professionals. J Gen Intern Med. 2009;24(12):1318-1321. DOI:10.1007/s11606-009-1129-z
