# Supplementary material for: East Meets West: A Multisite Validity Study of the China Medical Professionalism Inventory
Source: Perspect Med Educ. 2025 Sep 25;14(1):603–18. doi: 10.5334/pme.1682 (PMC12466328; doi:10.5334/pme.1682)
Supplement: Appendix 2. — China Medical Professionalism Inventory (CMPI) in English and Chinese, 20 items. [file pme-14-1-1682-s2.pdf]

Appendix 2 China Medical Professionalism Inventory (CMPI) in English and Chinese, 20 items

China Medical Professionalism Inventory (CMPI) in English, 20 items

**No.**	**Please check how often you have demonstrated the following behaviors in practice:**	**Not at all**	<---------------->			**Very much**
**1**	Presents professional opinions to the patient in a way the patient can understand.	1	2	3	4	5
**2**	Explains to the patient what they need to know about their problems, how and why they occurred, and what to expect next.	1	2	3	4	5
**3**	Demonstrates respect for patient autonomy by ensuring patients understand their situation and make informed clinical decisions	1	2	3	4	5
**4**	Discusses options with patients, asks their opinions, offers choices, and lets them decide what to do before making decisions.	1	2	3	4	5
**5**	Follows the patient's preference to accept or refuse any clinical treatment.	1	2	3	4	5
**6**	Maintains a positive rapport with the whole healthcare team and provides emotional support for colleagues.	1	2	3	4	5
**7**	Resolves interdisciplinary conflicts in a collegial and respectful manner.	1	2	3	4	5
**8**	Avoids discussing and revealing confidential patient information in public.	1	2	3	4	5
**9**	Maintains patient/physician relationships that do not exploit personal financial gain, privacy, or sexual advantages.	1	2	3	4	5
**10**	Avoids conducting non-scientific or unethical research supported by commercial sponsorship.	1	2	3	4	5
**11**	Explains treatment risks to patients fully and does not give patients false hope.	1	2	3	4	5
**12**	Provides appropriate and clear information to colleagues for follow-up patient care.	1	2	3	4	5
**13**	Applies new clinical practice guidelines into patient care actively and independently.	1	2	3	4	5

**14**	Acknowledges the meaning and relative value of scientific evidence in decision-making.	1	2	3	4	5
**15**	Consults other medical colleagues to manage a situation that is beyond one's ability.	1	2	3	4	5
**16**	Shares experience, skills, and knowledge with junior colleagues.	1	2	3	4	5
**17**	Pays attention to the risk factors that may threaten the safety of the patient by actively providing early warning and improvement suggestions to the relevant authority.	1	2	3	4	5
**18**	Does not provide unnecessary or excessive testing or medical treatment.	1	2	3	4	5
**19**	Ensures that the patient understands the content and meaning of informed consent correctly and fully.	1	2	3	4	5
**20**	Distinguishes between accepted treatment and experimental activities and abides by ethical standards.	1	2	3	4	5

China Medical Professionalism Inventory (CMPI) in Chinese, 20 Items
中国医师职业精神评价指标体系，20 个条目

**序号**	**请对如下行为的实现程度进行打分**	**完全不能**	<------------------>			**完全能够**
**1**	用患者可以理解的语言向他们解释专业判断（包括病情、诊断和治疗等）	1	2	3	4	5
**2**	向患者解释下一步的治疗计划，以及可能发生的情况及原因	1	2	3	4	5
**3**	能够尊重患者的自主权，确保他们在知情的前提下做出临床决策	1	2	3	4	5
**4**	在进行医疗决策前，充分听取患者意见，向患者解释可选择的治疗方案，辅助他们进行决策	1	2	3	4	5
**5**	能够尊重患者接受或拒绝任何医疗建议的权利	1	2	3	4	5
**6**	能够与团队成员保持良好人际关系并对同事给予情感上的支持	1	2	3	4	5

**7**	能够以合作和尊重的态度解决跨专业之间的矛盾	1	2	3	4	5
**8**	避免在公共场合讨论或公开涉及患者隐私或识别身份的信息	1	2	3	4	5
**9**	划清与患者利益界限，保持与患者之间的恰当关系，避免涉及个人财务利益、隐私和性交易	1	2	3	4	5
**10**	不得因医药企业的资助而进行有悖科学和伦理的研究	1	2	3	4	5
**11**	充分向患者说明治疗可能带来的风险，不给错误的希望或者假象	1	2	3	4	5
**12**	向同事移交患者进行后续治疗时，为同事提供必要的、足够的、清楚的患者信息	1	2	3	4	5
**13**	主动地在临床实践中应用最新临床指南	1	2	3	4	5
**14**	认识到在临床决策中使用循证医学的意义和价值	1	2	3	4	5
**15**	当遇到超出个人解决能力范围的问题时，能够向其他同事咨询或寻求帮助	1	2	3	4	5
**16**	将自己的技术和知识无私地传授给年轻或下级医师	1	2	3	4	5
**17**	时刻关注在临床实践中应可能威胁患者安全的危险因素，并积极向管理者提出危险预警和改进建议	1	2	3	4	5
**18**	不提供不必要的检测和治疗手段，不过度医疗	1	2	3	4	5
**19**	确保患者正确和充分地理解知情同意书的内容与含义，重视与患者关于知情同意的沟通	1	2	3	4	5
**20**	严格区分治疗行为与实验行为，恪守伦理道德准则	1	2	3	4	5
