# Supplementary material for: East Meets West: A Multisite Validity Study of the China Medical Professionalism Inventory
Source: Perspect Med Educ. 2025 Sep 25;14(1):603–18. doi: 10.5334/pme.1682 (PMC12466328; doi:10.5334/pme.1682)
Supplement: Appendix 3. — Interfactor correlations and Cronbach’s alpha coefficients of the 20-item China Medical Professionalism Inventory (CMPI). [file pme-14-1-1682-s3.pdf]

Appendix 3 Interfactor correlations and Cronbach's alpha coefficients of the 20-item China Medical Professionalism Inventory (CMPI)*

CMPI factors	n[†]	M (SD)	Interfactor Correlations: Pearson *r* (Cronbach's alpha)[a]			
			RCC	Int	Exc	Res
Respect, Compassion, and Communication	7	4.41 (0.47)	(0.88)	—	—	—
Integrity	5	4.50 (0.49)	0.61[b]	(0.90)	—	—
Excellence	4	4.43 (0.49)	0.53[b]	0.72[b]	(0.86)	—
Responsibility	4	4.45 (0.49)	0.57[b]	0.75[b]	0.82[b]	(0.87)

Abbreviations: RCC indicates respect, compassion, and communication; Int, integrity; Exc, excellence; Res, responsibility; M, mean; SD, standard deviation.

* Factors determined by the 3rd psychometric study of the CMPI (803 physicians). The number of physicians represents respondents with missing data removed—the number of surveys used for confirmatory factor analysis (CFA).

[†] n= the number of items in each of the four factors

[a] Cronbach's alpha values are those in parentheses on the diagonal.

[b] $P < .01$.
