# Supplementary material for: East Meets West: A Multisite Validity Study of the China Medical Professionalism Inventory
Source: Perspect Med Educ. 2025 Sep 25;14(1):603–18. doi: 10.5334/pme.1682 (PMC12466328; doi:10.5334/pme.1682)
Supplement: Appendix 4. — Summary of Items Included and Excluded from Chinese Policies and Experts. [file pme-14-1-1682-s4.pdf]

Appendix 4 Summary of Items Included and Excluded from Chinese Policies and Experts

**Process Resulting in Change to Item Pool**	**CMPI Version 1**	**CMPI Version 2 (n)** [a]	**CMPI Version 4 (n)** [b]	**CMPI (n)** [c]	**Summary** [d]
Topics of Chinese policy items added in Phase I					
Total	28	17	11	3	
Avoiding conflict of interest	6	5	4	1	#31
Providing appropriate medical treatment	2	2	2	1	#50
Avoiding risk and providing for patient safety	2	2	2	1	#49
Having responsibility for public health	3	1	1	0	
Maintaining access to care and not refusing to treat	1	1	1	0	
Providing appropriate care when patients dies	1	1	1	0	
Maintaining responsibility toward patients	4	1	0	0	
Assessing and reporting errors and misconduct	3	1	0	0	
Maintaining humanism and ethical standards	3	1	0	0	
Maintaining clinical excellence, guidelines	2	1	0	0	
Respecting patient autonomy and decisions	1	1	0	0	
Expert items added in Phase II					
Total	N/A[a]	2	2	2	
Commitment to excellence	N/A[a]	1	1	1	# 48
Respect patient autonomy	N/A[a]	1	1	1	#8
Total Policy + Expert added	28	19	13	5	
Removed policy items (each item's survey #)[$]		N/A [e]	#15, #18, #30, #36, #46, #54	#10, #13, #32, #33, #34, #52, #56, #58	

n is number of items (i.e., how many items in this column).

[a] Expert items added as part of the Phase II first expert survey.

[b] Policy items removed because of CITC, CAID, and EFA in Phase II first and second psychometric studies.

[c] Policy items removed as part of the Phase II second expert survey.

[d] Items retained in the final CMPI. Item numbers (indicated with #) correspond to the item numbers from the 58-item CMPI Version 2, the first psychometric study.

[e] We indicate N/A because Table 1 only provides CMPI Version 2; thus, one is not able to see items lost from Version 1 to Version 2

[$] Items "lost" from the prior version to this step. Items lost (indicated with #) correspond to the item numbers from the 58-item CMPI Version 2, the first psychometric study.
