# Supplementary material for: East Meets West: A Multisite Validity Study of the China Medical Professionalism Inventory
Source: Perspect Med Educ. 2025 Sep 25;14(1):603–18. doi: 10.5334/pme.1682 (PMC12466328; doi:10.5334/pme.1682)
Supplement: Appendix 5. — Characteristics of Existing Instruments. [file pme-14-1-1682-s5.pdf]

Appendix 5 Characteristics of Existing Instruments*

Instrument	Number of citations †	Content assessed by instrument	Number of items	Response options	Administration / Generalizability ‡	Instrument's factor	Theoretical foundation
**Directly Observed Behavioral Instruments (11 tools):**							
Simulation (OSCE)							
ECFMG®-CSA® [1]	118	Professionalism behavior	10 SP stations	A 4-point Likert scale (1=poor, 4=excellent)	SPs assess the candidates (OSCE)	Interviewing and collecting information; Counseling and delivering information; Rapport; Attentiveness; Personal manner	Professionalism as defined by the ABIM and the AAMC
p-OSCE [2]	15	Professionalism behavior	6 SP stations	A 3-point Likert scale (3=pass; 2=borderline; 1=fail)	SPs assess the candidates (OSCE)	Altruism, trust, and patient interest; Patient autonomy; Social justice	Professionalism as defined by the ACGME
Multi-Source Feedback Instruments							
GMC patient and colleague questionnaire [3]	109	Professional performance of doctors	Patient survey (8), colleague survey (25)	Binary options (yes/no)	Patients and colleagues assess physicians in real situation / 0.75 (36) for patients; 0.76 (12) for colleagues	Not described	Good medical practice as defined by GMC
p-360◦ evaluation [4]	27	Professionalism behavior	6	A 3-point Likert scale (3=pass; 2=borderline; 1=fail)	One nurse, two physicians, one peer, and one patient for each resident	Altruism, trust, and patient interest; Patient autonomy; Social justice	ACGME competencies
Direct Observation Instruments							
UMDSPAI [5]	63	Performance of professionalism in surgical residents	15	A 7-point continuous ordinal scale (with differing behavioral anchors)	Faculty assess residents and residents' self-evaluate in real situations	Punctuality; Appearance; Honesty / accountability / response to error; Compulsiveness; Responsibility / sense of duty; Response to criticism; Confidence / ability to assess oneself; Respect for others; Initiative / self-regulation; Altruism; Doctor-patient or doctor-family relationships; Inter-professional relationships; Trustworthiness / confidentiality; Moral and ethical standards; Attitude toward medical profession	ACGME competencies
P-MEX [6]	295	Professionalism behavior in medical practice	24	A 4-point Likert scale (1=unacceptable; 4=exceeded expectations; and not applicable)	Evaluators assess the residents in real situations / 0.80 (10-12)	Doctor-patient relationship skills; Reflective skills; Time management, and Interprofessional relationship skills	Literature review and experts committee meetings
EPRO-GP instrument [7]	86	Professional behavior in general practice	127	A 4-point Likert scale (1=hardly never, 4= almost always)	Self-assessment and trainers' assessment in real situations	Professionalism toward the patient; Professionalism toward other professionals; Professionalism toward the public; Professionalism toward oneself.	Literature review and experts committee meetings
Nijmegen Professionalism Scale [8]	52	Professional behavior of general practitioners	106	A 4-point Likert scale (1=never, 4=always)	Trainers assess trainees and trainees' self-assessment in real situations	Respecting patient's interests and professional distance; Collaboration skills and management skills; Responsibility and quality management; Reflection and learning and dealing with emotions.	EPRO-GP instrument [7]
Adaptation of AACS for foreign medical graduates [9]	23	Professional behavior of foreigner medical graduates	25	A 7-point Likert scale, ranges from 4 to 10 (7=being the expected level of performance)	Supervisors, residents, and nurses assess the graduates	Courteousness; Communication; Collaboration; Dedication; Self-appraisal	Literature review and expert committee meetings

p-mini-CEX [10]	15	Professionalism behaviors of dental interns	6	A 3-point Likert scale (3=pass; 2=borderline; 1=fail)	One attending physician assesses dental interns	Responsibility to patients; Respect for patients	P-MEX developed by Cruess et al. [5]
Peer Assessment Instrument							
Cottrell's peer assessment [11]	41	Professionalism performance	9	A 4-point Likert scale (1=too little, 4=too much) and "0" for not observe	Medical students assess medical students / 0.7(13)	Honesty and integrity; Accountability; Responsibility; Respectful and nonjudgmental behavior; Compassion and empathy; Maturity; Skillful communication; Confidentiality and privacy in all patient affairs; Self-directed learning; Appraisal skills	Literature review and codes were developed by the medical school curriculum committee
**English language instruments applied in Asia outside of China (5 tools)**							
Patient assessment of medical professionalism (Japan)[12]	8	Assessing medical students' professionalism from the patients' perspectives	11	A 5-point Likert scale (1=poor, 5= excellent)	Patients assess medical students	involvement and respect, compassion and rapport	Previous published paper[13]
Professionalism and ethics questionnaire (Malaysia)[14]	36	perceived unethical and unprofessional behavior	12	Disagree Neutral Agree	Medical students' self-assessment	Discipline, Plagiarism and cheating, Sexual harassment	None.
Medical Student Safety Attitudes and Professionalism Survey (Korea)[15]	43	perceptions with regard to patient safety	24	A 5-point Likert scale (1=strongly disagree, 5= completely agree)	surveys administered in face-to-face interviews	Safety culture, Teamwork culture, Error disclosure culture, Safety behavioural intent,	Previous published paper[16]
Salam A's medical professionalism assessment scale (Malaysia)[17]	48	Professionalism perspectives among medical students	27	A 5-point Likert scale (1=strongly disagree, 5= completely agree)	Medical students' self-assessment	Honesty, Accountability, Confidentiality, Responsibility	Literature review
Medical Professional Evaluation Scale (Japan) [18]	2	Professionalism performance among medical students	30	A 5-point Likert scale (1=strongly disagree, 5= completely agree)	Medical students' self-assessment	Level 1:Building interpersonal relationships, Planned learning, Interest in community health, Reflective practice, Knowledge and skills, Ethical and social responsibility and Self-management; Level 2:Providing safe, quality care, Providing patient-centered care, Planned learning, Collaborative practice, Building interpersonal relationships, Interest in community health, Ethical and social responsibility and Reflective practice	Literature review
**Chinese language instruments applied in China [¥] (9 tools):**							
Beijing Medical Residents Professionalism Attitude Scale [19]	6	Agreement of the description of professionalism	16	A 5-point Likert scale (1=I do not agree at all, 5=I agree completely)	Medical residents' self-assessment	Not described	Chinese Physician Charter
Chinese Penn State College of Medicine	3	Attitude towards professionalism behaviors and	27	A 5-point Likert scale (1= do not agree at all, 5= agree completely)	Physicians' self-assessment	Accountability; Altruism; Lifelong learning; Honesty and integrity; Respectful	Penn State College of Medicine

Professionalism Questionnaire [20]		beliefs					Professionalism Questionnaire [21]
Physician, nurse and patient perspectives on the meaning of professionalism [22]	19	Importance of professionalism behaviors	69	A 4-point Likert scale (1= not important at all, 4= very important)	Physicians' self-assessment, patient assessment of physicians, nurses' assessment of physicians	Not described	Previously published questionnaire [23]
Professionalism Competency Scale [24]	11	General description of physician professionalism	41	A 5-point Likert scale (1=very bad, 5= very good)	Experts assess physicians by general descriptions (not directed toward individuals)	Patients first; Patient autonomy; Social justice	Chinese physician charter
Medical Professionalism scale [25]	11	Agreement with professionalism items	21	A 5-point Likert scale (1=do not agree at all, 5= agree completely)	Physicians' self-assessment	Patient and physician communication skills; Accountability; Good communication; Teamwork	Literature review
Medical Professionalism Assessment Scale [26]	12	Importance of each item for professionalism	41	A 3-point Likert scale (1= very important, 3=not important)	Physicians' self-assessment	Honesty and integrity; Accountability; Lifelong learning; Skillful communication; Responsibility; Knowledgeable; Innovative skill; Respectful; Compassion and empathy; Altruism; Teamwork spirit	Literature review
Medical Professionalism Evaluation Index [27]	11	Importance of each item for professionalism	14	A 5-point Likert scale (1=I do not agree at all, 5=I agree completely)	Physicians' self-assessment	Moral value; Behavior; Attitude	Chinese Physician Charter
360-degree scale of professionalism for medical residents [28]	3	Performance of professionalism	68	A 5-point Likert scale (1=not satisfactory at all, 5=satisfactory completely)	Assessment of medical residents by managers, nurses, peers, faculty, and patients; Self reports by medical residents	Professionalism; Communication skills; System-based practice; Patient care	Previously published questionnaire [23]
Medical Residents Professionalism Assessment Scale [29]	3	Performance of professionalism	29	A 5-point Likert scale (1=very bad, 5=very good)	Medical residents' self-report; Faculty assessment of medical residents	Altruism; Honor and integrity; Caring, compassion and good communication; Respect for others; Conscientiousness; Pursuit of excellence; Leadership skills	Professionalism as defined by GMER, AAMCE, and ACGME
**Instruments with Top Validity Evidence * (3 tools):**							
Hisar's instrument for nursing students [30]	75	Professionalism attitudes of nursing students in Turkey	28	A 5-point Likert scale (1=I do not agree at all, 5=I agree completely)	Nursing students' self-assessment	Contribution to the increase of scientific information load; Autonomy; Cooperation; Competence, continuous education; Participation in professional organizations and professional development; Working in committees; Community service; Ethical codes and theory	Literature review
Nurse Practitioners' Roles and	24	Roles and competencies of	54	A 5-point Likert scale (1=completely disagree,	Nurses' self-assessment	Professionalism; Direct care; Clinical research; Practical guidance; Medical assistance; Leadership	Previous published paper [32]

Competencies **Scale** [31]		nurse practitioners		5=completely agree)		and reform	
Perceived Faculty Competency Inventory [33]	33	Faculty Competence	33	A 5-point Likert scale (1=strongly disagree, 5= completely agree)	Clinical, counseling, and school psychology students' assessment of their faculty	Professionalism / ethics; Clinical supervision; Research; Multicultural competence; Advising / mentoring	Literature review

* This table was adapted and reprinted with permission of PLOS ONE from the original paper: Li H, Ding N, Zhang Y, Liu Y, Wen D. Assessing medical professionalism: a systematic review of instruments and their measurement properties. PloS one. 2017 May 12;12(5):e0177321.
[†] Number of citations in Google Scholar or in China's Wanfang Data Base as of April 16, 2025.
[‡] Number of surveys required to obtain an equivalent dependability coefficient. Numbers outside the parentheses are the dependability coefficient. Numbers inside the parentheses are number of surveys required.
[¥] These Chinese language instruments do not have formal English names. These descriptions of instruments were translated from Chinese into English by author Li, H.

References List

1. van Zanten M, Boulet JR, Norcini JJ, et al. Using a standardised patient assessment to measure professional attributes. Med Educ. 2005;39(1):20-29.
2. Yang YY, Lee FY, Hsu HC, et al. Validation of the behavior and concept-based assessment of professionalism competence in postgraduate first-year residents. J China Med Assoc. 2013; 76: 186–194.
3. Campbell JL, Richards SH, Dickens A, et al. Assessing the professional performance of UK doctors: an evaluation of the utility of the General Medical Council patient and colleague questionnaires. Qual Saf Health Care. 2008;17(3):187-193.
4. Yang YY, Lee FY, Hsu HC, et al. Assessment of first-year post-graduate residents: usefulness of multiple tools. J Chin Med Assoc. 2011; 74:531-538.
5. Gauger PG, Gruppen LD, Minter RM, et al. Initial use of a novel instrument to measure professionalism in surgical residents. Am J Surg. 2005;189(4):479-487.
6. Cruess R, Mcllroy JH, Cruess S, et al. The professionalism mini-evaluation exercise: A preliminary investigation. Acad Med. 2006; 81: S74–S78.
7. Van de Camp K, Vernooij-Dassen M, Grol R, et al. Professionalism in general practice: Development of an instrument to assess professional behaviour in general practitioner trainees. Med Educ. 2006; 40: 43–50.
8. Tromp F, Vernooij-Dassen M, Kramer A, et al. Behavioural elements of professionalism: assessment of a fundamental concept in medical care. Med Teach. 2010;32(4):e161-e169.
9. Tromp F, Rademakers JJ, Ten Cate TJ. Development of an instrument to assess professional behaviour of foreign medical graduates. Med Teach. 2007;29(2-3):150-155.
10. Yang YY, Hsu HC, Lee FY, et al. Evaluation of the clinical competences of dental interns with mini-CEX. J Med Educ. 2010; 14:251-261.
11. Cottrell S, Diaz S, Cather A, et al. Assessing medical student professionalism: An analysis of a peer assessment. Med Educ Online. 2006; 11: 1-8.
12. Fujikawa H, Son D, Aoki T, Kondo K, Takemura Y, Saito M, Den N, Eto M. Translating and validating a Japanese version of the instrument for patient assessment of medical professionalism (J-IPAMP): a cross-sectional survey. BMC Med Educ. 2022 Aug 23;22(1):641. doi: 10.1186/s12909-022-03699-8.
13. Ratelle JT, Halvorsen AJ, Mandrekar J, Sawatsky AP, Reed DA, Beckman TJ. Internal medicine resident professionalism assessments: exploring the association with patients' overall satisfaction with their hospital stay. Acad Med. 2020;95(6):902‑10.
14. Yadav H, Jegasothy R, Ramakrishnappa S, Mohanraj J, Senan P. Unethical behavior and professionalism among medical students in a private medical university in Malaysia. BMC Med Educ. 2019;19(1):218.
15. Lee H-Y, Hahm M-I, Lee SG. Undergraduate medical students' perceptions and intentions regarding patient safety during clinical clerkship. BMC Med Educ. 2018;18(1):66.
16. Liao JM, Etchegaray JM, Williams ST, Berger DH, Bell SK, Thomas EJ. Assessing medical students' perceptions of patient safety: the medical student safety attitudes and professionalism survey. Acad Med. 2014;89(2):343–51
17. Haque M, Zulkifli Z, Haque SZ, Kamal ZM, Salam A, Bhagat V, Alattraqchi AG, Rahman NIA. Professionalism perspectives among medical students of a novel medical graduate school in Malaysia. Adv Med Educ Pract. 2016;7:407-22.
18. Yamamoto T, Kawaguchi A, Otsuka Y. Developing the comprehensive medical professionalism assessment scale. MedEdPub. 2019;8(91):91.
19. Yue SF, Chen J, Jiang GC, et al. Investigation of and Research on Medical Professionalism Cognition of Residents in Beijing Hospitals. Chin Cont Med Educ. 2018,10(10):8-11.[in Chinese]
20. Song WW, Shi L, Li HH, et al. The introduction of the Penn State College of Medicine Professionalism Questionnaire and the evaluation on its validity and reliability. Chin J Med Educ. 2019,39(11):868-871. [in Chinese]
21. Blackall G F, Melnick S A, Shoop G H, et al. Professionalism in medical education: the development and validation of a survey instrument to assess attitudes toward professionalism. Med Teach. 2007, 29(2-3): e58-e62.
22. Chen K, Zhao M, Li RF, et al. Investigation on the Connotation of Medical Professionalism from the Perspective of Physician, Nurse and Patient. Med and Phil. 2015(3):40-44.[in Chinese]
23. Green M, Zick A, Makoul G. Defining professionalism from the perspective of patients, physicians, and nurses. Acad Med. 2009, 84(5): 566-573.
24. Tian L, Zhao YH, Li JG, et al. Evaluation on medical professionalism from the construction of physicians' competency model. Chin J of Med Educ Res. 2012,11(6):553-557.[in Chinese]
25. Huo YL, Zhou FF. Medical Professionalism Scale Preliminary Preparation and Structure Exploration. Med and Phil. 2015,36(19):27-30.[in Chinese]
26. Xu X, Yuan HY. A research on designing the Physician Professionalism Evaluation Scale. Chin Hos Mange. 2015,35(12):50-52.[in Chinese]
27. Xu X, Yuan HY. Research on the Construction of Evaluation Index System for Physician Professionalism---Based on 8 Three-A Hospitals in Shanghai. Chin Med Ethics. 2016,29(2):195-198.[in Chinese]
28. Chen K, Ma BF, Zhao M. Research on 360-degree Assessment Method Measuring Medical Professionalism and Communication Skills of Residents. Med and Phil. 2017,38(1):71-74,80.[in Chinese]
29. Li R, Li YP, Peng J. The analysis and evaluation of resident professionalism. Chin J Med Educ. 2016,36(2):287-290.[in Chinese]
30. Hisar F, Karadag A, Kan A. Development of an instrument to measure professional attitudes in nursing students in Turkey. Nurse Educ Today. 2010; 30: 726–730.
31. Lin LC, Lee S, Ueng SW, et al. Reliability and validity of the Nurse Practitioners' Roles and Competencies Scale. J Clin Nurs. 2016; 25: 99–108.
32. Hawkins RE, Katsufrakis PJ, Holtman MC, et al. Assessment of medical professionalism: who, what, when, where, how, and … why? Med Teach. 2009;31(4):348–361.
33. Deemer ED, Thomas D, Hill CL. Measuring students' perceptions of faculty competence in professional psychology: Development of the Perceived Faculty Competence Inventory. Train Educ Prof Psychol. 2011; 5: 38–47.
